# Hydroxysafflor yellow A attenuates sepsis-induced intestinal barrier dysfunction by modulating Bcl-2/SOD2-mediated mitochondrial apoptosis

**DOI:** 10.3389/fphar.2026.1728183

**Published:** 2026-02-02

**Authors:** Jinzhong Fei, Chencheng Xu, Chaochao Chen, Qing Chen, Zhengbin Wu, Yaoli Wang, Daiqin Bao, Shifeng Shao

**Affiliations:** 1 Department of ICU, Daping Hospital, Army Medical University, Chongqing, China; 2 Department of Post-Graduate School, Army Medical University, Chongqing, China; 3 The 955 Hospital of the Chinese People’s Liberation Army Ground Force, Changdu, China; 4 Department of Anesthesiology, Daping Hospital, Army Medical University, Chongqing, China

**Keywords:** apoptosis, Bcl-2, hydroxysafflor yellow A, sepsis-induced intestinal barrier dysfunction, SOD2

## Abstract

**Background:**

Sepsis remains a major cause of hospital mortality. Sepsis-induced intestinal injury is regarded as the driving force behind the rapid progression of critical conditions such as shock and sepsis, and serves as the initiating factor of subsequent organ dysfunction. Therefore, the development of effective therapeutic agents to restore intestinal barrier function is crucial for improving outcomes in sepsis.

**Methods:**

A caecal ligation and puncture (CLP) model was established in mice to induce sepsis, and intestinal epithelial cells (IEC-6) were treated with lipopolysaccharide (LPS) to simulate sepsis *in vitro*. These models were used to investigate the protective efficacy and molecular mechanisms of hydroxysafflor yellow A (HSYA) against sepsis-induced intestinal barrier dysfunction.

**Results:**

HSYA alleviated intestinal barrier dysfunction in septic mice, markedly reduced levels of inflammatory factors, and improved survival. *In vitro*, HSYA enhanced barrier function of IECs, reduced mitochondrial fragmentation and reactive oxygen species (ROS) accumulation, promoted proliferation and inhibited apoptosis by upregulating the expression of Bcl-2 and SOD2.

**Conclusion:**

The study demonstrated the therapeutic potential and underlying mechanisms of HSYA in ameliorating sepsis-induced intestinal barrier injury, providing a new strategy for sepsis treatment.

## 1 Introduction

Sepsis, defined as life-threatening organ dysfunction caused by a dysregulated host response to infection, continues to pose a severe global health burden. Epidemiological data indicate that more than 49 million sepsis cases occur worldwide each year, with mortality rates approaching 20%, leading to extensive healthcare resource consumption and posing a critical public health challenge (Rudd et al., 2020; Fleischmann et al., 2016). Among the complex pathological processes of sepsis, intestinal barrier dysfunction has been identified as a pivotal driver of disease progression (Ren et al., 2024; Meng et al., 2017). Systemic inflammatory storms, oxidative stress, and microcirculatory impairment collectively destabilize intestinal epithelial tight junction proteins like occludin, claudin-1, and ZO-1, while also inducing excessive apoptosis of intestinal mucosal cells (Horowitz et al., 2023; Sturgeon and Fasano, 2016; Zhang et al., 2022; Mohammad and Thiemermann, 2020). These pathological changes increase intestinal permeability, promote bacterial and endotoxin translocation, and perpetuate systemic inflammation and multi-organ failure. Restoring intestinal barrier integrity has emerged as a central therapeutic strategy for sepsis.

Xuebijing injection, a representative agent in integrative sepsis therapy, has been validated in multicenter clinical studies to significantly reduce 28-day mortality and alleviate intestinal barrier dysfunction (Liu et al., 2023). Its mechanisms include suppression of TLR4/NF-κB–mediated inflammatory cascades, improvement of microcirculatory disturbances, and mitigation of oxidative stress (Zhou W. et al., 2021; Zhang et al., 2025). Hydroxysafflor yellow A (HSYA), the principal active constituent of Xuebijing, is a water-soluble chalcone glycoside extracted from *Carthamus tinctorius* (safflower). Recent studies have demonstrated that HSYA alone exerts marked organ-protective effects in various disease models. In models of myocardial ischemia–reperfusion, HSYA activates the PI3K/Akt axis, suppresses mitochondrial-dependent apoptosis, downregulates Bax, upregulates Bcl-2, and prevents caspase-3 activation (Liang et al., 2024). In acute lung injury, HSYA restores mitochondrial membrane potential, enhances the functions of superoxide dismutase (SOD) and glutathione peroxidase, and reduces mitochondrial reactive oxygen species (ROS) accumulation (Sun et al., 2010). Despite these findings, the role of HSYA in sepsis and its underlying mechanisms remains insufficiently characterized.

Mitochondria, as central regulators of energy metabolism and apoptosis, play a critical role in sepsis-induced intestinal barrier injury. In septic conditions, intestinal epithelial mitochondria undergo structural damage and functional disruption, including impaired ATP synthesis, ROS overproduction, and mitochondrial DNA injury (Kong et al., 2022; Xu et al., 2021). Insufficient energy supply diminishes epithelial repair capacity, whereas excessive ROS disrupts tight junction complexes (Otani et al., 2020). Furthermore, the abnormal opening of the mitochondrial permeability transition pore promotes cytochrome c release, triggering the caspase-9/caspase-3 apoptotic cascade and accelerating epithelial cell death (Wang W. et al., 2025). Persistent activation of this mitochondria-apoptosis axis ultimately compromises both mechanical and biological intestinal barriers. Although HSYA has been widely recognized for its anti-inflammatory, antioxidant, and anti-apoptotic activities, its contribution to sepsis-associated intestinal barrier injury has not been comprehensively explored. The present study employed septic mice and lipopolysaccharide (LPS)-stimulated intestinal epithelial cells (IECs) to evaluate the therapeutic potential of HSYA against intestinal barrier dysfunction. In addition, network pharmacology was applied to uncover potential molecular targets and signaling pathways. This study showed that HSYA, as a major monomeric component of Xuebijing, exerts protective effects on intestinal barrier function independently, highlighting its potential as a candidate for novel sepsis therapeutics.

## 2 Methods

### 2.1 Data collection and processing

The chemical structure of HSYA was retrieved from the PubChem database (https://pubchem.ncbi.nlm.nih.gov/). Using this structural information, potential targets were predicted using Prediction (https://prediction.charite.de/subpages/target_prediction.php), PharmMapper (http://lilab-ecust.cn/pharmmapper/), and BATMAN-TCM (http://bionet.ncpsb.org.cn/batman-tcm/). The predicted targets were then converted into their corresponding gene symbols through the UniProt database (http://www.uniprot.org/), thereby generating the HSYA-related dataset.

### 2.2 Functional enrichment analysis

To further explore the biological relevance of the HSYA-associated targets, Gene Ontology (GO) and Kyoto Encyclopedia of Genes and Genomes (KEGG) enrichment analyses were performed. GO annotations encompassed three categories: biological processes (BP), molecular functions (MF), and cellular components (CC). KEGG pathway mapping was carried out to associate these targets with key signaling pathways and integrate them into broader genomic contexts. All enrichment analyses were carried out utilizing the R packages clusterProfiler and GOplot (version 1.0.2), which provide robust and widely accepted analytical frameworks for functional characterization.

### 2.3 Molecular docking analysis

Molecular docking was performed with the Glide module in the Schrödinger software suite, applying both Standard Precision (SP) and Extra Precision (XP) algorithms. HSYA was docked into the crystal structure of TP53 (PDB code: 3D06). The XP docking mode was used to predict the most stable binding conformation and to elucidate key interactions between HSYA and its binding pocket.

### 2.4 Molecular dynamics (MD) simulation

To further examine the docking results, MD simulations of the HSYA–TP53 complex were performed with the Desmond module of the Schrödinger package. The system was solvated, neutralized with counter-ions, and energy minimized with the OPLS3 force field. After a preliminary 1 ns equilibration run, a 100 ns production simulation was conducted under NPT conditions at 300 K. The simulation trajectory was analyzed for root mean square deviation (RMSD), root mean square fluctuation (RMSF), ligand–protein contact mapping, and binding profile characterization to assess the stability and interaction dynamics of the complex.

### 2.5 Animal model

Adult male C57BL/6 mice (8–10 weeks old, 20–22 g) were provided by the Laboratory Animal Center of the Army Medical University, Chongqing, China. All experimental procedures were approved by the Ethics Committee of the Army Medical University (Approval No. AMUWEC20257048) and performed in compliance with the ARRIVE guidelines and the principles of the Basel Declaration. The animals were supplied under Production License No. SCXK (Yu) 2022-0011 and Use License No. SYXK (Yu) 2022-0018.

Experimental sepsis was established by cecal ligation and puncture (CLP). Mice were anesthetized via intraperitoneal injection of sodium pentobarbital (30 mg/kg). After loss of pain reflexes, animals were fixed on a surgical board, disinfected with povidone-iodine, and a 1 cm midline incision was created to expose the cecum. In the Sham group, only laparotomy and cecal exposure were performed without ligation or puncture.

For the Sepsis (Sep), HSYA, HSYA + 2-ME, and HSYA + ABT-263 groups, the cecum was ligated approximately one-quarter of its length below the ileocecal valve with sterile 4-0 silk sutures. A single puncture was made in the distal cecum with a 22-gauge needle, and ∼0.2 mL of fecal material was gently extruded to confirm patency. The cecum was repositioned, and the abdominal wall was closed in two layers with sterile 2-0 sutures. Animals were housed individually for postoperative observation. Twelve hours after CLP, mean arterial pressure (MAP) was measured via carotid artery cannulation, and a ≥30% decrease in MAP was used as the criterion for successful model establishment. Mice in the Sep group received no further treatment. The HSYA group was given HSYA (50 mg/kg, intraperitoneal) 1 h before CLP. The HSYA + 2-ME group received HSYA (50 mg/kg, intraperitoneal) 1 h before CLP and 2-ME (10 mg/kg, intraperitoneal) 1 h after CLP. The HSYA + ABT-263 group received HSYA (50 mg/kg, intraperitoneal) 1 h before CLP and navitoclax (10 mg/kg, intraperitoneal) 1 h after CLP. Tissue samples were collected 24 h postoperatively for subsequent analyses.

### 2.6 Cell culture and reagents

#### 2.6.1 IEC-6 cell culture

The rat intestinal epithelial cell line IEC-6 (ATCC, USA) was maintained in Dulbecco’s Modified Eagle Medium (DMEM; Hyclone, USA) supplemented with 10% fetal bovine serum (FBS; Hyclone, USA) under standard conditions (37 °C, 5% CO_2_, humidified incubator). The IECs were grown to 30%–50% confluence and transfected with shRNA adenovirus to knock down the expression levels of Bcl2 and SOD2 at a multiplicity of infection (MOI) of 50 (Supplementary Table S1). After the virus transfection, this study replaced Opti MEM containing adenovirus with complete culture medium until the cell confluence reached 75%–80%. IEC-6 cells were allocated into five experimental groups: (i) Control (CTL), cultured in serum-free DMEM for 12 h; (ii) LPS, treated with 1 μg/mL LPS (Sigma, USA) for 12 h; (iii) HSYA, treated with 1 μg/mL LPS and then treated with 20 μM HSYA for 24 h (iv) LPS + Si-Bcl2, the expression of Bcl2 was knock down with Bcl2-shRNA adenovirus transfection and then treated with 1 μg/mL LPS for 12 h and 20 μM HSYA for 24 h sequentially. (v) LPS + Si-SOD2, the expression of SOD2 was knock down with SOD2-shRNA adenovirus transfection and then treated with 1 μg/mL LPS for 12 h and 20 μM HSYA for 24 h sequentially.

#### 2.6.2 Mitochondrial oxygen consumption rate detection

The oxygen consumption rate (OCR) was measured using a 24-well XFe plate from Seahorse, Agilent, USA. IECs were seeded at 2 × 10^4^ cells per well and treated as illustrated above. IECs were prepared for 1 h in assay medium, followed by sequential addition of 2 μM oligomycin, 1 μM FCCP, and 0.5 μM rotenone/antimycin A. The OCR was measured with an extracellular flux analyzer to induce mitochondrial stress.

#### 2.6.3 Reagents

HSYA (S9601) was obtained from Selleck. Navitoclax (HY-10087) and 2-ME (HY-12033) were purchased from MCE. ELISA kits for DAO (E-EL-M0412), D-LAC (E-BC-K002-M), LPS (E-EL-0180), TNF-α (E-EL-M3063), IL-6 (E-EL-M0044), and IL-1β (E-EL-M0037) were supplied by Elabscience (China). DCFH-DA fluorescent probe (S0033S), MitoTracker Red CMXRos (C1049B), Ki67 cell proliferation detection kit (C2312S), TUNEL apoptosis detection kit (C1088), and ROS detection kit (S0033S) were supplied by Beyotime Biotechnology (Shanghai, China). Cell Counting Kit-8 (CCK-8, HY-K0301) was purchased from MedChemExpress (USA). Primary antibodies against SOD2 (ab118340), Bcl-2 (ab194583), β-actin (ab8226), and ZO-1 (ab307799) were obtained from Abcam (USA).

### 2.7 Histopathological analysis of intestinal tissues (hematoxylin–eosin, HE staining)

At the indicated experimental time points, mice were euthanized with an intraperitoneal overdose of sodium pentobarbital. Approximately 10 cm of proximal small intestine adjacent to the stomach was excised, rinsed with cold PBS to remove fecal contents, and fixed in 4% paraformaldehyde for 48–72 h. Fixed tissues were cut into 2–3 mm fragments, dehydrated, cleared, paraffin-embedded, and sectioned. Sections were stained with HE, mounted, and examined under a light microscope to assess histopathological changes.

### 2.8 Transmission electron microscopy (TEM)

At the same sampling time, mice were euthanized with an overdose of sodium pentobarbital, and approximately 10 cm of proximal small intestine near the stomach was excised. After fecal removal by alternating washes with PBS and sodium citrate, both ends of the intestinal segment were ligated, and 2.5% glutaraldehyde was perfused into the lumen for 20 min. The tissue was cut into 1–2 mm fragments and immersed in 2.5% glutaraldehyde at room temperature (RT) for 30 min, followed by 2 h in sucrose at 4 °C. Samples were post-fixed in osmium tetroxide for 2 h in the dark, dehydrated in graded acetone, and embedded in epoxy resin. Polymerization was sequentially performed at RT (1.5 h), 37 °C (24 h), and 60 °C (48 h). Ultrathin sections were stained with uranyl acetate and lead citrate, and ultrastructural changes in intestinal epithelial cells were examined utilizing a TEM (JEM-1400, JEOL, Japan).

### 2.9 Western blotting

Proteins were extracted from intestinal tissues or IEC-6 cells using RIPA buffer supplemented with protease inhibitors, and concentrations were determined with a BCA assay. Equal protein amounts were resolved by SDS–PAGE and transferred to PVDF membranes. After blocking with 5% nonfat milk at RT for 2 h, membranes were incubated overnight at 4 °C with the following primary antibodies: SOD2 (1:1,000), Bcl-2 (1:1,000), ZO-1 (1:1,000), and β-actin (1:5,000). Following extensive washing, membranes were probed with goat anti-rabbit secondary antibody (1 : 20,000) for 1 h at RT. Protein bands were visualized using the ChemiDoc, and densitometric analysis was performed with ImageJ software.

### 2.10 Immunofluorescence assay

Following experimental treatments, IEC-6 cells were fixed with 4% paraformaldehyde for 10 min and rinsed three times with PBS (5 min each wash). Cells were permeabilized with 0.3% Triton X-100 for 1 min at RT, then blocked in 1% BSA for 1 h. Samples were incubated overnight at 4 °C with anti–ZO-1 primary antibody (1 : 200), followed by a fluorescent secondary antibody (1 : 200) for 1 h at RT in the dark. Nuclei were counterstained with DAPI, and images were acquired using a laser scanning confocal microscope.

### 2.11 Animal survival analysis

Sixteen mice were randomly assigned to each group using a random number table. Survival time and survival rate were monitored and recorded over 72 h, starting at 12 h after CLP surgery.

### 2.12 Statistical analysis

All statistical analyses were performed using R software (version 4.0.2, https://www.r-project.org/). Normally distributed continuous data were compared using independent Student’s t-tests, while non-normally distributed data were analyzed with the Mann–Whitney U test (Wilcoxon rank-sum test). ROC curves were constructed with the pROC package, and statistical significance was defined as *P* < 0.05 (two-tailed).

## 3 Results

### 3.1 HSYA attenuates sepsis-induced intestinal injury

**FIGURE 1:**
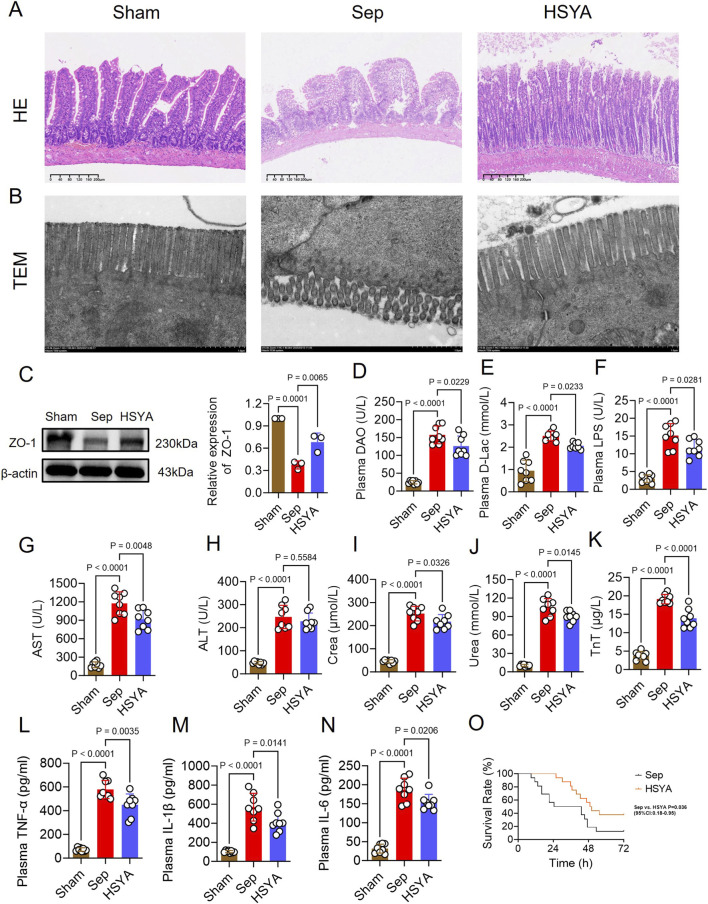
HSYA protects mice against sepsis-induced intestinal injury. **(A,B)** Representative HE-stained sections and TEM images of intestinal tissues (HE: scale bar = 40 μm; TEM: scale bar = 1 μm). **(C)** Western blot analysis of ZO-1 expression (n = 3). **(D–N)** Serum levels of DAO, D-lac, LPS, ALT, AST, creatinine, urea, troponin T, TNF-α, IL-1β, and IL-6 (n = 8). **(O)** Survival analysis of septic mice after HSYA treatment (n = 16).

Histopathological analysis revealed distinct differences between groups. In Sham mice, HE staining showed tightly arranged villi, continuous epithelial layers, intact crypts, and no obvious inflammatory infiltration (Figure 1A). In contrast, septic mice exhibited severe pathological changes, including shortened and fused villi with rupture and epithelial shedding, crypt destruction, extensive neutrophil and lymphocyte infiltration, and marked stromal edema (Figure 1A). HSYA treatment ameliorated these abnormalities, restoring villus height, reducing epithelial shedding, alleviating inflammatory infiltration, and preserving crypt morphology (Figure 1A). TEM further confirmed these findings (Figure 1B). Sham mice displayed dense and orderly microvilli with intact tight junctions, whereas septic mice showed sparse and fragmented microvilli, disrupted junctions, and widened intercellular spaces (Figure 1B). HSYA intervention improved microvillus density and alignment, maintained junctional continuity, and reduced mitochondrial swelling and endoplasmic reticulum dilation (Figure 1B). Tight junction integrity was further evaluated by ZO-1 expression. Western blotting revealed a 62.54% reduction in ZO-1 expression in intestinal tissues compared with Sham controls, while HSYA treatment increased ZO-1 expression by 82.3% relative to septic mice, indicating partial restoration of barrier integrity (Figure 1C).

Plasma biomarkers supported these observations. DAO, D-lac, and LPS levels were significantly elevated in septic mice, increasing 5.4-, 1.8-, and 4-fold compared with Sham controls. HSYA reduced these levels by 20.76%, 18.02%, and 23%, respectively (Figures 1D–F). In addition, markers of hepatic, renal, and myocardial injury were improved. Serum AST, ALT, creatinine, urea, and troponin T were decreased by 7.61%, 21.97%, 14.11%, 14.28%, and 27.5% in the HSYA group, respectively, compared with septic mice (Figures 1G–K).

Systemic inflammation was also attenuated. Serum TNF-α, IL-6, and IL-1β were reduced by 22.44%, 28%, and 17.6% in the HSYA group, respectively, relative to septic mice (Figures 1L–N). Importantly, HSYA improved survival outcomes. In the Sepsis group, most mice died within 24 h, with only two surviving beyond 72 h, resulting in a 12.5% survival rate and a mean survival time of 33.75 h. In contrast, HSYA treatment increased the 72 h survival rate to 37.5% and prolonged the mean survival time to 49.51 h (Figure 1O). Together, these findings demonstrate that HSYA mitigates sepsis-induced intestinal structural damage, restores barrier integrity, reduces systemic inflammation, and improves survival in septic mice.

### 3.2 HSYA attenuates LPS-induced IECs apoptosis and improved mitochondrial function

**FIGURE 2:**
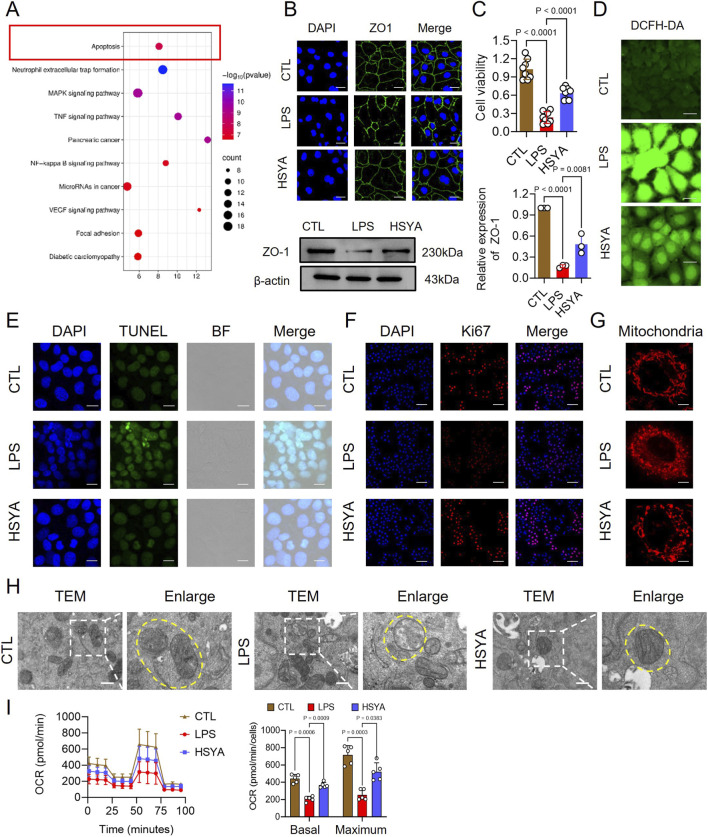
Proteomic Analysis for Identifying Key Molecules Mediating Ferroptosis in Sepsis-Associated IEC-6 Cells **(A)** KEGG pathway enrichment analysis. **(B)** Immunofluorescence staining of ZO-1 (scale bar = 50 μm) and Western blot analysis of ZO-1 expression. **(C)** CCK-8 assay of cell viability (n = 8). **(D)** DCFH-DA showing ROS levels (scale bar = 20 μm, n = 8) **(E)** TUNEL staining of apoptotic cells. **(F)** Ki-67 immunofluorescence of proliferating cells **(G)** MitoTracker Red CMXRos staining showing mitochondrial morphology (scale bar = 50 μm, n = 3). **(H)** TEM images of mitochondrial ultrastructure. **(I)** Impact of LPS on mitochondrial respiration in IEC-6 cells (n = 3).

This study then collected normal and LPS-stimulated IECs for transcriptomics detection, followed by a differential analysis. Transcriptomics suggested that intestinal epithelial cells underwent obvious apoptosis following LPS treatment (Figure 2A). Immunofluorescence staining demonstrated that ZO-1 localization at the cell borders was markedly disrupted and fragmented in LPS-treated IEC-6 cells, indicating severe tight junction damage. In contrast, HSYA treatment restored continuous and compact linear ZO-1 staining along the cell membrane, suggesting stabilization of the ZO-1 complex and recovery of barrier integrity. Western blotting also confirmed this conclusion (Figure 2B). CCK-8 assays revealed that LPS reduced cell viability, whereas HSYA increased viability by 1.7-fold compared with the LPS group (Figure 2C).

We further detected the effect of HSYA on LPS-induced IECs apoptosis. Considering the central role of mitochondrial dysfunction in initiating oxidative stress and apoptosis, intracellular ROS generation were further evaluated. DCFH-DA staining demonstrated that ROS fluorescence intensity in the LPS-treated group was elevated by approximately 2.03-fold relative to controls, whereas HSYA treatment reduced ROS levels by 19.34% (Figure 2D; Supplementary Figure S1A). TUNEL staining showed abundant granular green fluorescent nuclei in LPS-treated cells, reflecting extensive apoptosis, while HSYA markedly reduced TUNEL-positive cells and fluorescence intensity (Figure 2E). Consistently, Ki-67 staining demonstrated impaired proliferation following LPS treatment, whereas HSYA significantly restored the proportion of proliferating cells (Figure 2F). Intracellular mitochondrial integrity was further evaluated, quantitative analysis of mitochondrial morphology indicated that HSYA increased the proportion of elongated mitochondria by 51.39% and reduced the proportion of fragmented mitochondria by 30.57% relative to LPS-treated cells (Figure 2G; Supplementary Figure S1B). TEM further confirmed these findings: LPS-treated cells displayed swollen mitochondria with focal matrix rarefaction, vacuolization, and disrupted cristae, whereas HSYA treatment preserved mitochondrial ultrastructure with dense matrix and intact lamellar cristae (Figure 2H). Meanwhile, this study found that HSYA significantly elevated oxygen consumption rate, improving mitochondrial function (Figure 2I).

Collectively, these results demonstrate that HSYA mitigates mitochondrial injury, reduces oxidative stress, inhibits apoptosis, and promotes epithelial repair in LPS-stimulated IEC-6 cells.

### 3.3 HSYA inhibited mitochondrial mediated apoptosis by up-regulating BCL2 and SOD2

**FIGURE 3:**
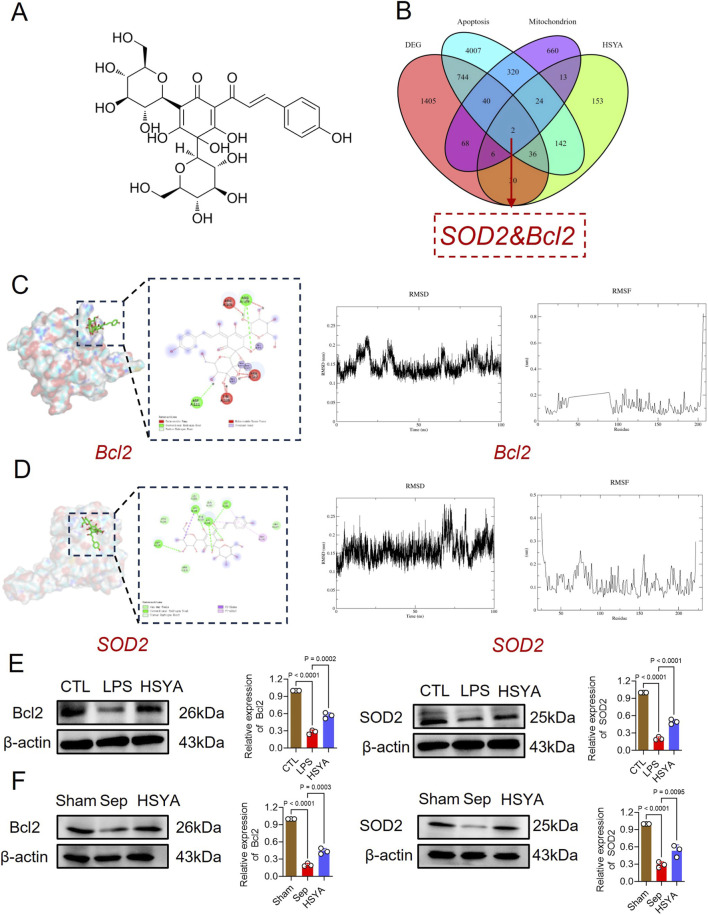
Molecular Docking, Molecular Dynamics Analysis, and *in vitro*/*in vivo* Validation of Key Proteins. **(A)** Chemical structure of HSYA. **(B)** Venn diagram integrating sepsis-, HSYA-, and apoptosis-related targets, highlighting Bcl-2 and SOD2 as key molecules. **(C)** Molecular docking and molecular dynamics analysis showing binding interactions of HSYA with Bcl-2. **(D)** Molecular docking and molecular dynamics analysis showing binding interactions of HSYA with SOD2. **(E)** Western blot analysis of Bcl-2 and SOD2 protein expression in IEC-6 cells (n = 3). **(F)** Western blot analysis of Bcl-2 and SOD2 protein expression in intestinal tissues (n = 3).

To elucidate the mechanisms by which HSYA restores intestinal barrier function in sepsis, network pharmacology analysis was performed. The chemical structure of HSYA is presented in Figure 3A. This study first retrieved 406 potential targets sites of HSYA based on the Swiss Target Prediction database. We used Venn analysis to intersect the differential genes from transcriptomics, HSYA’s target sites, mitochondria-associated genes and apoptosis-related genes. The targets B-cell lymphoma-2 (Bcl-2) and Superoxide dismutase (SOD2) were finally obtained (Figure 3B).

To obtain deeper insights into the molecular mechanisms by which HSYA mitigates sepsis-induced intestinal barrier disruption, molecular docking and molecular dynamics simulation were performed to assess its binding with Bcl-2 and SOD2 (Figures 3C,D). These findings suggest that HSYA forms stable complexes with both Bcl-2 and SOD2. The Bcl-2 complex exhibits stronger binding stability, whereas the SOD2 complex maintains structural equilibrium.

Western blot analysis was employed to detect the effect of HSYA on the expression of Bcl-2 and SOD2 both *in vivo* and *in vitro*. WB results showed that the expression of Bcl-2 and SOD2 was significantly downregulated in IECs and intestinal tissues following sepsis, whereas HSYA could significantly recover the expression of Bcl-2 and SOD2 (Figures 3E,F).

### 3.4 HSYA inhibited mitochondrial mediated apoptosis by up-regulating Bcl-2

**FIGURE 4:**
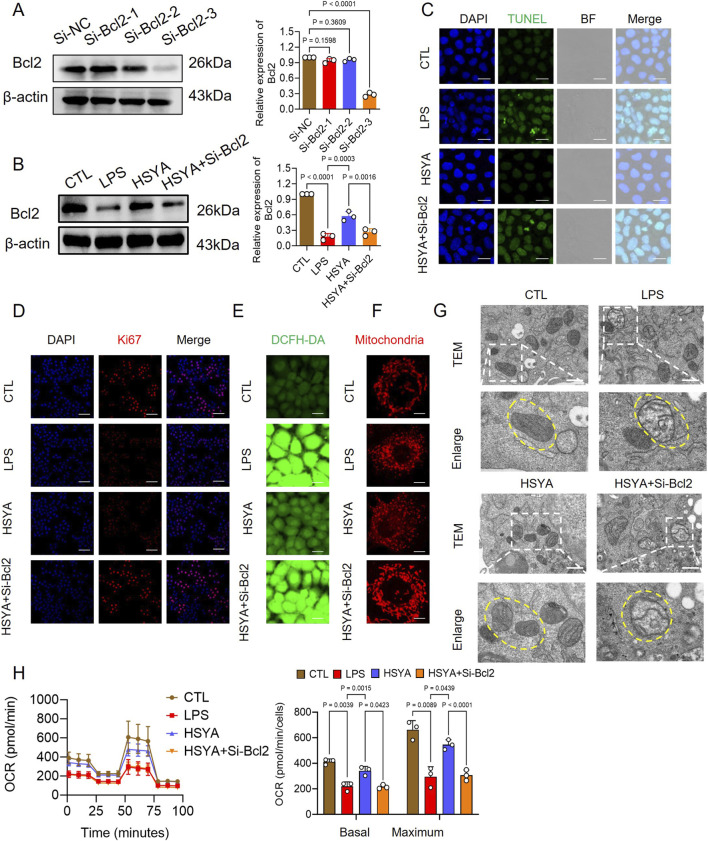
*In vitro* effects of HSYA on IEC-6 cells following Bcl-2 inhibition. **(A)**Western blot analysis showing the relative expression level of Bcl-2 after treatment in Si-Bcl-2 of IEC-6 cells (n = 3). **(B)** Western blot analysis showing the effects of HSYA + Si-Bcl2 on the expression of Bcl2 in LPS-stimulated IEC-6 cells (n = 3). **(C)** TUNEL staining of apoptotic cells (scale bar = 50 μm, n = 3). **(D)** Ki-67 immunofluorescence for proliferating cells. **(E,F)** DCFH-DA (n = 3) and MitoTracker Red CMXRos staining (n = 8) showing intracellular ROS levels and mitochondrial morphology in IEC-6 cells (scale bar = 20 μm). **(G)** TEM images showing mitochondrial ultrastructure in IEC-6 cells following HSYA and HSYA + Si-Bcl-2 treatment. **(H)** Impact of HSYA + Si-Bcl-2 treatment on mitochondrial respiration in LPS-stimulated IEC-6 cells (n = 3).

To verify whether HSYA attenuates mitochondrial-mediated apoptosis in IECs following sepsis based on regulation of the expression of Bcl-2, *in vitro*, we performed experiments by knocking down the expression of Bcl-2 in IECs with Bcl-2-shRNA adenovirus transfection. WB results showed that Bcl-2-shRNA adenovirus significantly reduced the expression of Bcl-2 in IECs and antagonized the recovery of cell viability following HSYA treatment (Figures 4A,B and; Supplementary Figure S2A). Subsequently, we detected the effects on apoptosis level and mitochondrial function. The result demonstrated that compared with the HSYA -treated group, the knockdown of Bcl-2 could upregulate IECs apoptosis. Compared with the HSYA group, knockdown of Bcl-2 significantly enhanced apoptotic signals with stronger TUNEL (Figure 4C) and intracellular ROS fluorescence (Figure 4E and; Supplementary Figure S2B) and less Ki-67 positivity rate (Figure 4D). Meanwhile, the knockdown of Bcl-2 could damage the mitochondrial morphology, leading to mitochondrial fragmentation (Figure 4F; Supplementary Figure S2C). TEM further demonstrated that the knockdown of Bcl-2 could damage the mitochondrial morphology, including spherical swelling, reduced matrix electron density, and disorganized cristae (Figure 4G). Furthermore, seahorse results showed that the knockdown of Bcl-2 also could damage the mitochondrial dysfunction, causing lower OCR rate (Figure 4H). Eventually, we examined the effect of knocking down the expression of Bcl-2 in IECs on barrier function. The results of immunofluorescence and WB showed that knocking down of Bcl-2 antagonized the recovery of ZO-1 in IECs following HSYA treatment (Supplementary Figures S2D, S2E).

**FIGURE 5:**
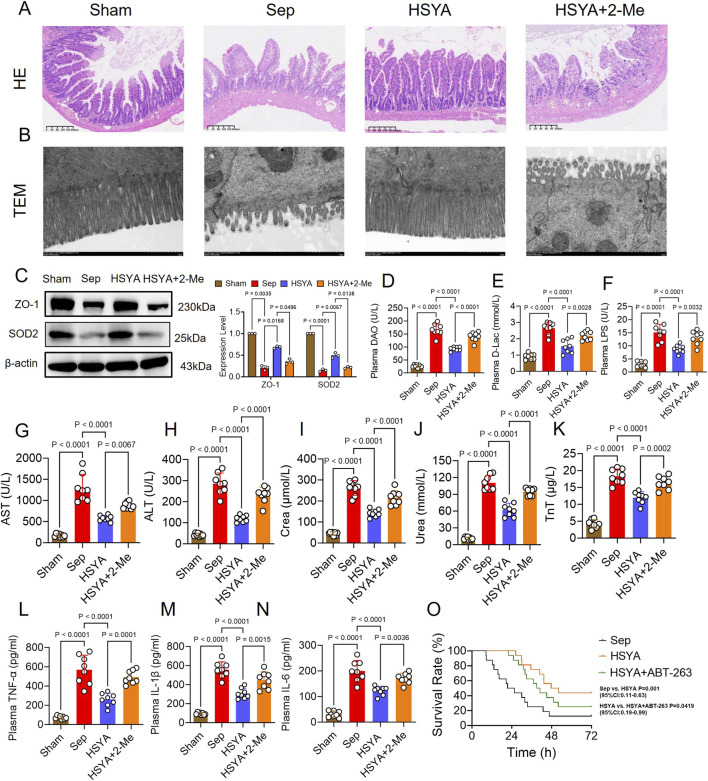
Effects of HSYA on sepsis-induced intestinal injury in mice through Bcl-2 upregulation. **(A,B)** Representative HE-stained sections and TEM images of intestinal tissues (HE: scale bar = 40 μm; TEM: scale bar = 1 μm). **(C)** Western blot analysis of ZO-1 protein expression in intestinal tissues (n = 3). **(D–N)** Serum levels of DAO, D-lac, LPS, ALT, AST, creatinine, urea, troponin T, TNF-α, IL-1β, and IL-6 (n = 8). **(O)** Survival analysis of septic mice after HSYA treatment (n = 16).

Furthermore, we validated in an *in vivo* model using the specific Bcl-2 inhibitor ABT-263 to downregulate the expression of Bcl-2 and detected the effect on sepsis-induced intestinal barrier dysfunction. HE staining revealed that ABT-263 caused pronounced villus defects and goblet cell reduction, characterized by shortened and edematous villi with diminished lymphocyte infiltration, compared with the HSYA group (Figure 5A). TEM further confirmed these observations (Figure 5B). Meanwhile, this study found that the expression of ZO-1 and Bcl-2 in the intestinal tissues was weakened due to ABT-263 (Figures 5C,D). DAO, D-lac, and LPS levels were significantly reduced by 25.63%, 44.31%, and 34.67% in the HSYA group compared with Sepsis controls (Figures 5D–F). Following ABT-263 administration, these markers increased by 26.11%, 48.44%, and 23.52%, respectively, relative to HSYA treatment (Figures 5D–F). These results of plasma biomarkers associated with intestinal barrier dysfunction further supported that ABT-263 reduced the effect of HSYA on the recovery of sepsis-induced intestinal barrier dysfunction.

Serum markers of hepatic, renal, and myocardial injury were also examined. HSYA treatment decreased AST, ALT, creatinine, urea, and troponin T by 32.90%, 50.64%, 32.74%, 24.96%, and 40.91%, respectively, compared with the Sepsis group. Inhibition of Bcl-2 with ABT-263 reversed these effects, resulting in increases of 23.2%, 62.94%, 42.09%, 17.64%, and 36.54%, respectively, compared with HSYA alone (Figures 5G–K). Inflammatory cytokine analysis revealed that HSYA significantly attenuated systemic inflammation. IL-1β, IL-6, and TNF-α levels were decreased by 50.68%, 40.88%, and 52.15% compared with the Sepsis group (Figures 5F–N). ABT-263 administration partially abolished these effects, with cytokine levels increasing by 61.6%, 47.20%, and 54.48%, respectively, relative to HSYA treatment. As for animal survival, in the sepsis group, most mice died within 24 h, with only 2 surviving beyond 72 h and a mean survival time of 23.55 h. HSYA treatment markedly improved outcomes, with 7 mice surviving beyond 72 h and a mean survival time of 49.94 h. In contrast, ABT-263 treatment reduced survival, with 4 mice surviving beyond 72 h and a mean survival time of 40 h (Figure 5O).

### 3.5 HSYA inhibited mitochondrial mediated apoptosis by up-regulating SOD2

**FIGURE 6:**
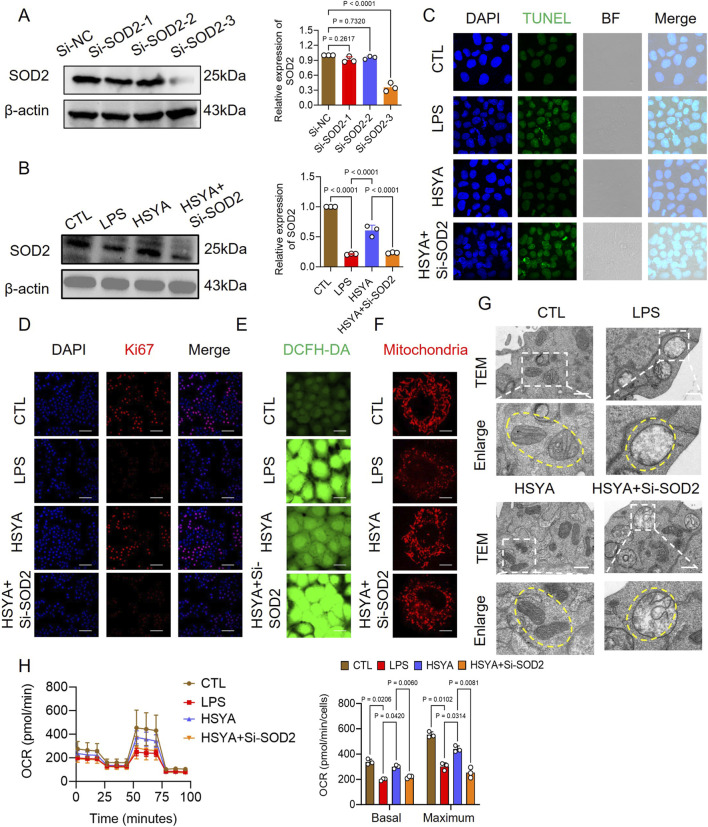
Effects of HSYA on intestinal epithelial cells under silence SOD-2. **(A,B)** Western blot analysis of SOD2 protein expression in IEC-6 cells (n = 3). **(C)** TUNEL staining for apoptotic cells (scale bar = 50 μm).**(D)** Ki-67 immunofluorescence for proliferating cells (scale bar = 50 μm, n = 8). **(E,F)** DCFH-DA (n = 8)and MitoTracker Red CMXRos staining (n = 3) showing intracellular ROS accumulation and mitochondrial morphology in IEC-6 cells (scale bar = 20 μm). **(G)** TEM images showing mitochondrial ultrastructure in IEC-6 cells treated with HSYA or HSYA + Si-SOD2 (scale bar = 1 μm). **(H)** Impact of HSYA + Si-SOD2 treatment on mitochondrial respiration in LPS-stimulated IEC-6 cells (n = 3).

Meanwhile, to verify the effect of SOD2 in the process of the therapy of HSYA on sepsis-induced IECs apoptosis, *in vitro*, we performed experiments by knocking down the expression of SOD2 in IECs with SOD2-shRNA adenovirus transfection. WB results showed that SOD2-shRNA adenovirus significantly reduced the expression of SOD2 in IECs and antagonized the recovery of cell viability following HSYA treatment (Figures 6A,B and; Supplementary Figure S3A). Subsequently, we found that compared with the HSYA -treated group, the knockdown of SOD2 could upregulate IECs apoptosis, damage mitochondrial morphology and function, and weaken IECs barrier function (Figures 6C–H; Supplementary Figure S3B–E).

**FIGURE 7:**
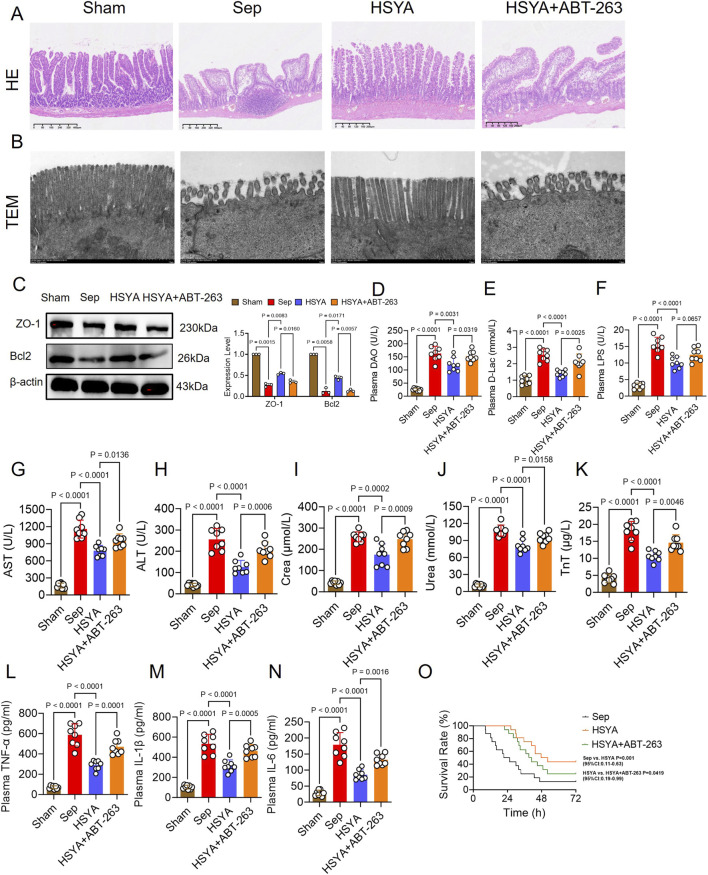
Effects of HSYA on sepsis-induced intestinal injury in mice following SOD2 inhibition. **(A,B)** Representative HE-stained sections and TEM images of intestinal tissues (HE: scale bar = 40 μm; TEM: scale bar = 1 μm). **(C)** Western blot analysis of ZO-1 protein expression in intestinal tissues (n = 3). **(D–N)** Serum levels of DAO, D-lac, LPS, ALT, AST, creatinine (Crea), urea, troponin T (TnT), TNF-α, IL-1β, and IL-6 (n = 8). **(O)** Kaplan–Meier survival curves showing the effects of HSYA treatment on sepsis-induced mortality in mice (n = 16).

**FIGURE 8:**
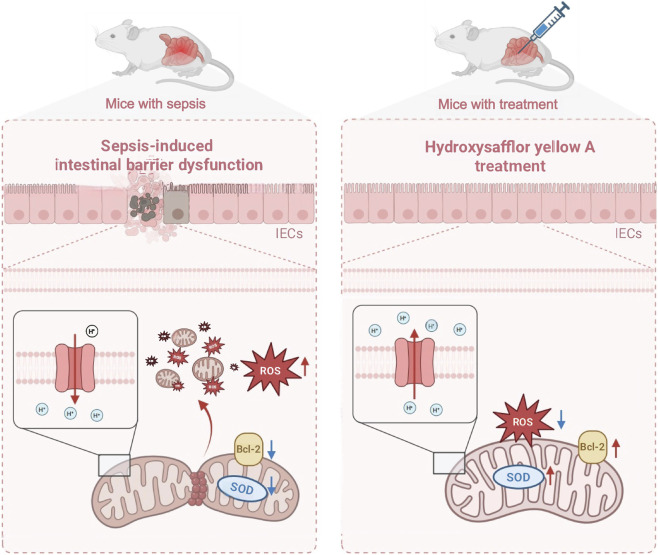
The mechanism diagram of the protective effects of HSYA on sepsis-induced intestinal injury. The red arrow pointing upward represents promotion, and the red arrow pointing downward represents inhibition.

Furthermore, we validated in an *in vivo* model using the specific SOD2 inhibitor 2-Me to downregulate the expression of SOD2 and detected the effect on sepsis-induced intestinal barrier dysfunction. HE staining and TEM results showed that 2-Me damaged intestinal barrier function compared with the HSYA group (Figures 7A,B). Meanwhile, this study found that the expression of ZO-1 and SOD2 in the intestinal tissues was also reduced with 2-Me administration compared with the HSYA group (Figure 7C). Serum markers related to intestinal barrier dysfunction including DAO, D-lac, and LPS levels showed that following 2-Me administration reduced the effect of HSYA on intestinal barrier function (Figures 7D,E). Serum markers of hepatic, renal, and myocardial injury and serum markers of inflammatory factors including IL-1β, IL-6, and TNF-α together demonstrated that 2-Me administration partially abolished organ function protection and anti-inflammatory effects of HSYA (Figures 7G–N). Animal survival analysis further confirmed these effects. In the Sepsis group, only two mice survived beyond 72 h, with a mean survival time of 23.29 h. HSYA treatment markedly improved survival, with all eight mice surviving 72 h and a mean survival time of 65.26 h. However, inhibition of SOD2 attenuated this benefit, as only four mice survived 72 h in the HSYA + 2-ME group, with a mean survival time of 40 h (Figure 7O). These results suggest that HSYA can reduce mitochondria-related apoptosis and oxidative stress-associated injury in intestinal after sepsis, which may be closely related to HSYA improving mitochondrial quality by regulating Bcl-2 and SOD-2 (Figure 8).

## 4 Discussion

This study provides the first evidence that the traditional Chinese medicine monomer HSYA exerts protective effects against sepsis-induced intestinal barrier dysfunction and elucidates its underlying molecular mechanisms. In a CLP-induced murine model, HSYA significantly attenuated intestinal barrier injury, reduced systemic inflammation, and improved survival. At the cellular level, LPS-treated intestinal epithelial cells exhibited marked apoptosis accompanied by profound mitochondrial structural and functional impairment. HSYA pretreatment preserved mitochondrial integrity, restored bioenergetic function, and markedly reduced apoptosis. Mechanistic analysis further demonstrated that HSYA restored the expression of Bcl-2 and SOD2 suppressed by LPS, thereby limiting apoptosis and ameliorating intestinal barrier dysfunction during sepsis.

The intestinal barrier constitutes a key defense for maintaining host homeostasis, and its integrity depends on precisely coordinated structural and functional units. The mucosal surface is composed of villi and crypts, where stem cells at the crypt base continuously differentiate into multiple epithelial lineages. As the core of the physical barrier, the apical glycocalyx and tight junction complexes tightly regulate paracellular transport of luminal contents, preventing pathogen and toxin invasion (Sturgeon and Fasano, 2016). Under physiological conditions, the intestinal microbiota and its metabolites, especially short-chain fatty acids like butyrate, contribute to maintaining barrier integrity by sustaining epithelial energy metabolism, upregulating tight junction proteins like occludin and ZO-1, and limiting excessive inflammatory responses (Fu et al., 2019; Shen et al., 2021; Feng et al., 2018). However, during sepsis and other critical illnesses, systemic cytokine storms, oxidative stress, and ischemia/reperfusion injury converge on the epithelium. These insults induce the internalization and degradation of tight junction proteins, compromising barrier integrity (Odenwald and Turner, 2017; Pérez-Hernández et al., 2021), and simultaneously trigger profound mitochondrial dysfunction and oxidative stress within IECs, leading to the activation of intrinsic apoptotic pathways (Wang Y. et al., 2025). Clinical studies have reported strong correlations between oxidative stress biomarkers, such as malondialdehyde (MDA), and intestinal barrier injury markers, such as I-FABP, in sepsis patients (Lu et al., 2024; Coufal et al., 2020). Consequently, controlling oxidative stress and suppressing epithelial apoptosis represent central therapeutic strategies for repairing intestinal barrier injury in sepsis.

Mitochondria, the primary energy factories of the cell, play a central role in apoptosis mediated by mitochondrial dysfunction, which is a key driver of multiple organ dysfunction syndrome (MODS) in sepsis (Li et al., 2023). Under physiological conditions, mitochondria maintain homeostasis through continuous fission and fusion, while matrix antioxidant enzymes, such as SOD2, eliminate excess ROS to preserve structural and functional integrity (Court et al., 2024). However, systemic inflammation and oxidative stress during sepsis severely disrupt mitochondrial stability. On one hand, mitochondrial dynamics become imbalanced: excessive fission driven by Drp1 hyperactivation, combined with insufficient fusion caused by Mfn2 downregulation, leads to cristae disruption and mitochondrial fragmentation (Tábara et al., 2025; Hu et al., 2021). On the other hand, impairment of the electron transport chain (ETC) markedly increases ROS production, while reduced SOD2 expression compromises antioxidant defenses, further amplifying oxidative injury. Excessive ROS not only directly damages mitochondrial proteins, lipids, and mitochondrial DNA (mtDNA), but also promotes the cytoplasmic release of mtDNA, which subsequently activates the cGAS–STING pathway and amplifies inflammatory signaling (Zhou L. et al., 2021; Cheng et al., 2020). Mitochondrial dysfunction represents a fundamental mechanism underlying apoptosis and organ injury in sepsis. Clinical studies by Katharina G et al. reported that decreased mitochondrial membrane potential in immune cells of septic patients is strongly correlated with the severity of MODS (Star et al., 2023). Hence, therapeutic strategies aimed at modulating key anti-apoptotic regulators and antioxidant molecules to preserve mitochondrial function hold promise for alleviating sepsis-induced organ damage.

B-cell lymphoma 2 (BCL-2) is a mitochondrial membrane protein that regulates apoptosis by blocking Bax- and Bak-mediated oligomerization in the outer mitochondrial membrane (OMM), thereby preventing cytochrome c release and caspase activation (Spitz and Gavathiotis, 2022). Mechanistically, BCL-2 suppresses mitochondrial outer membrane permeabilization and limits cytochrome c efflux. In addition, BCL-2 interacts with the endoplasmic reticulum (ER) to regulate intracellular Ca^2+^ signaling, further contributing to the control of apoptosis (Loncke et al., 2021; de Ridder et al., 2023). By serving as cellular stress sensors, BH3-only proteins neutralize anti-apoptotic BCL-2 members upon activation, thereby freeing Bax and Bak to initiate apoptosis. Bax and Bak then undergo conformational changes, translocate from the cytosol to the OMM, and oligomerize to form pores that disrupt mitochondrial integrity. This process facilitates the release and activation of cytochrome c, triggering the downstream caspase cascade and ultimately leading to apoptosis. Bax and Bak also influence mitochondrial dynamics, including fission and fusion, underscoring their cooperative role in regulating both the stability and disruption of mitochondrial function during apoptosis (Vogler et al., 2025; Sandow et al., 2021; Schweighofer et al., 2024).

Previous studies have highlighted the role of BCL-2 in intestinal epithelial injury. Song et al. showed that in a necrotizing enterocolitis (NEC) mouse model and IEC-6 cells, CCL3 upregulation exacerbated intestinal damage by promoting apoptosis through the CCL3–CCR4–ERK1/2–NFκB–BAX/BCL-2 axis (Yuan et al., 2022). Zhi et al. employed a chemotherapy-induced diarrhea (CID) mouse model and an IEC-6 cell model, and reported that non-replicative, heat-killed Bifidobacterium conferred protective effects by modulating the BCL–2–mediated mitochondrial apoptosis pathway, effectively alleviating diarrhea and suppressing intestinal epithelial apoptosis (Yan et al., 2025). Our study indicates that HSYA attenuated sepsis-induced intestinal barrier dysfunction by upregulating BCL-2 and limiting mitochondria-mediated apoptosis in IECs.

SOD is a key antioxidant enzyme that converts superoxide radicals into hydrogen peroxide, forming the first defense against mitochondrial oxidative damage. Among its isoforms, mitochondrial SOD2 plays an essential role in dismutating superoxide anions (O_2_^−^) to hydrogen peroxide (H_2_O_2_), thereby maintaining redox balance within the mitochondrial matrix. Previous studies have shown that SOD2 deficiency results in elevated oxidative stress, excessive ROS accumulation, impaired mitochondrial antioxidant defenses, and increased susceptibility to apoptosis (Gordon et al., 2022; Kasahara et al., 2005). For example, Peng et al. reported that miR-23a-5p aggravated intestinal ischemia–reperfusion injury in an IEC-6 hypoxia model by targeting PPARα and enhancing SOD2-related oxidative stress. Similarly, Lee et al. demonstrated that 5-aminosalicylic acid protected IEC-6 cells from NSAID-induced injury by scavenging free radicals and reducing SOD2-associated apoptosis (Jung et al., 2020).

In line with previous observations, our data demonstrated that HSYA treatment significantly increased SOD2 activity in the intestinal epithelial cells of septic mice. This upregulation was accompanied by a marked reduction in mitochondrial ROS levels. Importantly, increased SOD2 activity strongly correlated with improved mitochondrial morphology observed by TEM and reduced MDA content. These findings provide direct evidence that HSYA protects the intestinal epithelium by reinforcing SOD2-mediated mitochondrial antioxidant defenses, thereby attenuating oxidative stress–induced structural damage.

Recent international studies have further highlighted that intestinal barrier failure is not merely a downstream consequence of systemic inflammation, but can actively amplify septic progression. In this context, mitochondrial dysfunction is increasingly viewed as a central hub that couples inflammatory stress to epithelial injury and organ dysfunction. A recent review summarized that sepsis can impair oxidative phosphorylation, increase mitochondrial reactive oxygen species, disturb mitochondrial dynamics, and compromise mitochondrial quality control, thereby promoting tissue damage and multi-organ failure (Hu et al., 2024).

Oxidative stress is a key upstream driver of these mitochondrial lesions. A recent narrative review emphasized that reactive oxygen and nitrogen species disrupt mitochondrial integrity and redox balance and can intensify inflammatory signaling. The same review also discussed emerging antioxidant strategies that aim to restore redox homeostasis and improve outcomes in sepsis (Lin et al., 2025).

Recent HSYA-focused studies provide important benchmarks and help frame the novelty of our work. Chen and colleagues reported that HSYA alleviated sepsis-associated pulmonary and intestinal injury by inhibiting TP53-mediated ferroptosis, linking HSYA to regulated cell death pathways that influence barrier integrity (Chen et al., 2025). Pan and colleagues showed that HSYA improved multi-organ injury in a CLP model and proposed modulation of metabolic networks and the JAK2 and STAT1 pathway, further supporting that HSYA acts as a multi-target compound in sepsis (Pan et al., 2025). Together with these reports, our findings add a mitochondria-centered epithelial protection axis supported by functional perturbation, in which HSYA restores Bcl-2 and SOD2, improves mitochondrial quality, and attenuates oxidative stress-associated injury, ultimately contributing to intestinal barrier preservation.

Several limitations should be acknowledged. First, although genetic knockdown and pharmacological interference support functional involvement of BCL-2 and SOD2 in HSYA-mediated protection, definitive causality cannot be fully established from the current study design. Future work using sepsis models with intestinal epithelium–specific BCL-2 and/or SOD2 knockout will be important to confirm causality. In addition, because pharmacological inhibitors may exert off-target effects, complementary genetic approaches and direct target engagement assays will further strengthen mechanistic inference. Second, our mechanistic investigations were conducted in rat-derived IEC-6 cells, whereas the *in vivo* sepsis model was performed in mice. Species-specific differences in inflammatory signaling and drug responsiveness may influence effect size and should be addressed in future studies using mouse-derived primary intestinal epithelial cells or intestinal organoids. Third, the low intrinsic bioavailability of HSYA remains a challenge for clinical translation. Advanced delivery strategies, including nanotechnology-based liposomes and polymeric micelles, may improve intestinal targeting and enhance bioavailability. Finally, HSYA is a multi-target compound, and the BCL-2 and SOD2 axis likely represents a major, but not exclusive, mechanism. Other pathways relevant to sepsis and intestinal barrier protection may include activation of antioxidant programs such as Nrf2, suppression of inflammatory signaling such as NF-κB, regulation of alternative forms of regulated cell death such as ferroptosis, and modulation of mitochondrial quality control processes including mitophagy. Future studies assessing representative markers across these pathways will help delineate how these mechanisms interact with mitochondria-associated apoptosis and oxidative stress in mediating HSYA’s protective effects.

## Data Availability

The original contributions presented in the study are included in the article/Supplementary Material, further inquiries can be directed to the corresponding authors.
