# Correction: Hydroxysafflor yellow a attenuates sepsis-induced intestinal barrier dysfunction by modulating Bcl-2/SOD2-mediated mitochondrial apoptosis

**DOI:** 10.3389/fphar.2026.1806009

**Published:** 2026-03-05

**Authors:** Jinzhong Fei, Chencheng Xu, Chaochao Chen, Qing Chen, Zhengbin Wu, Yaoli Wang, Daiqin Bao, Shifeng Shao

**Affiliations:** 1 Department of ICU, Daping Hospital, Army Medical University, Chongqing, China; 2 Department of Post-Graduate School, Army Medical University, Chongqing, China; 3 The 955 Hospital of the Chinese People’s Liberation Army Ground Force, Changdu, China; 4 Department of Anesthesiology, Daping Hospital, Army Medical University, Chongqing, China

**Keywords:** apoptosis, Bcl-2, hydroxysafflor yellow a, sepsis-induced intestinal barrier dysfunction, SOD2

There was a mistake in Figure 6 as published. When preparing the TUNEL in Figure 6C, the TUNEL of Figure 4C was erroneously used as the paradigm. The corrected Figure 6 appears below.

**FIGURE 6:**
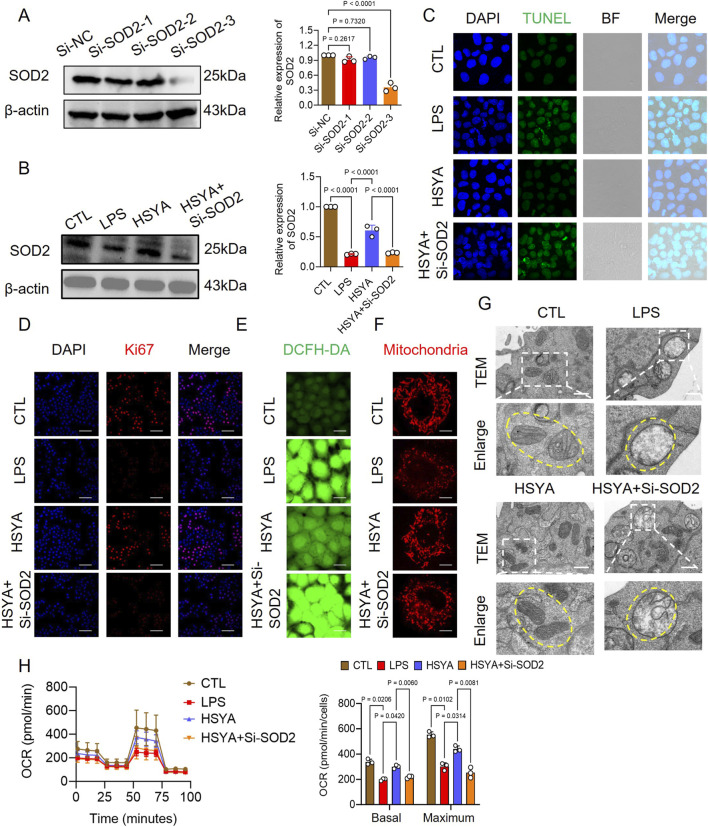
Effects of HSYA on intestinal epithelial cells under silence SOD-2. **(A,B)** Western blot analysis of SOD2 protein expression in IEC-6 cells (n = 3). **(C)** TUNEL staining for apoptotic cells (scale bar = 50 μm). **(D)** Ki-67 immunofluorescence for proliferating cells (scale bar = 50 μm, n = 8). **(E,F)** DCFH-DA (n = 8) and MitoTracker Red CMXRos staining (n = 3) showing intracellular ROS accumulation and mitochondrial morphology in IEC-6 cells (scale bar = 20 μm). **(G)** TEM images showing mitochondrial ultrastructure in IEC-6 cells treated with HSYA or HSYA + Si-SOD2 (scale bar = 1 μm). **(H)** Impact of HSYA + Si-SOD2 treatment on mitochondrial respiration in LPS-stimulated IEC-6 cells (n = 3).

The original article has been updated.

