# Supplementary figures and images for: Hydroxysafflor yellow A attenuates sepsis-induced intestinal barrier dysfunction by modulating Bcl-2/SOD2-mediated mitochondrial apoptosis
Source: Front Pharmacol. 2026 Feb 2;17:1728183. doi: 10.3389/fphar.2026.1728183 (PMC12907381; doi:10.3389/fphar.2026.1728183)

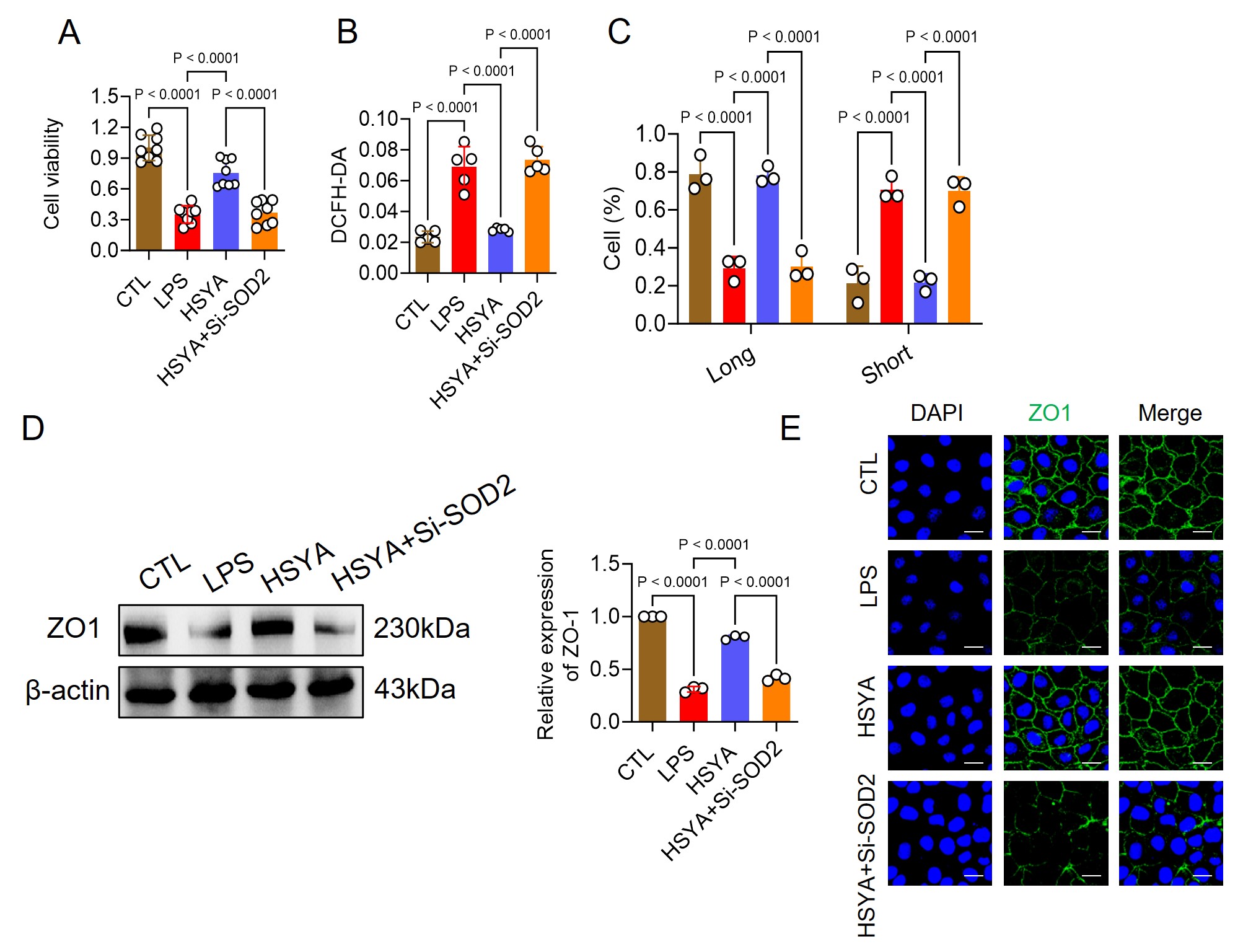

Supplement: Supplementary file 1 [file Image3.jpeg]

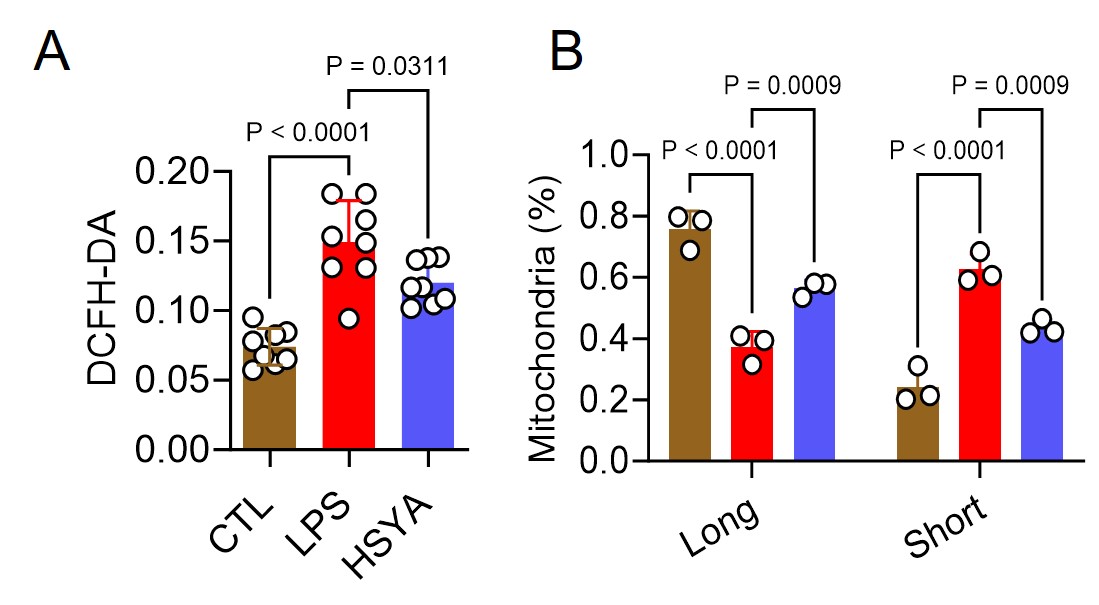

Supplement: Supplementary file 2 [file Image1.jpeg]

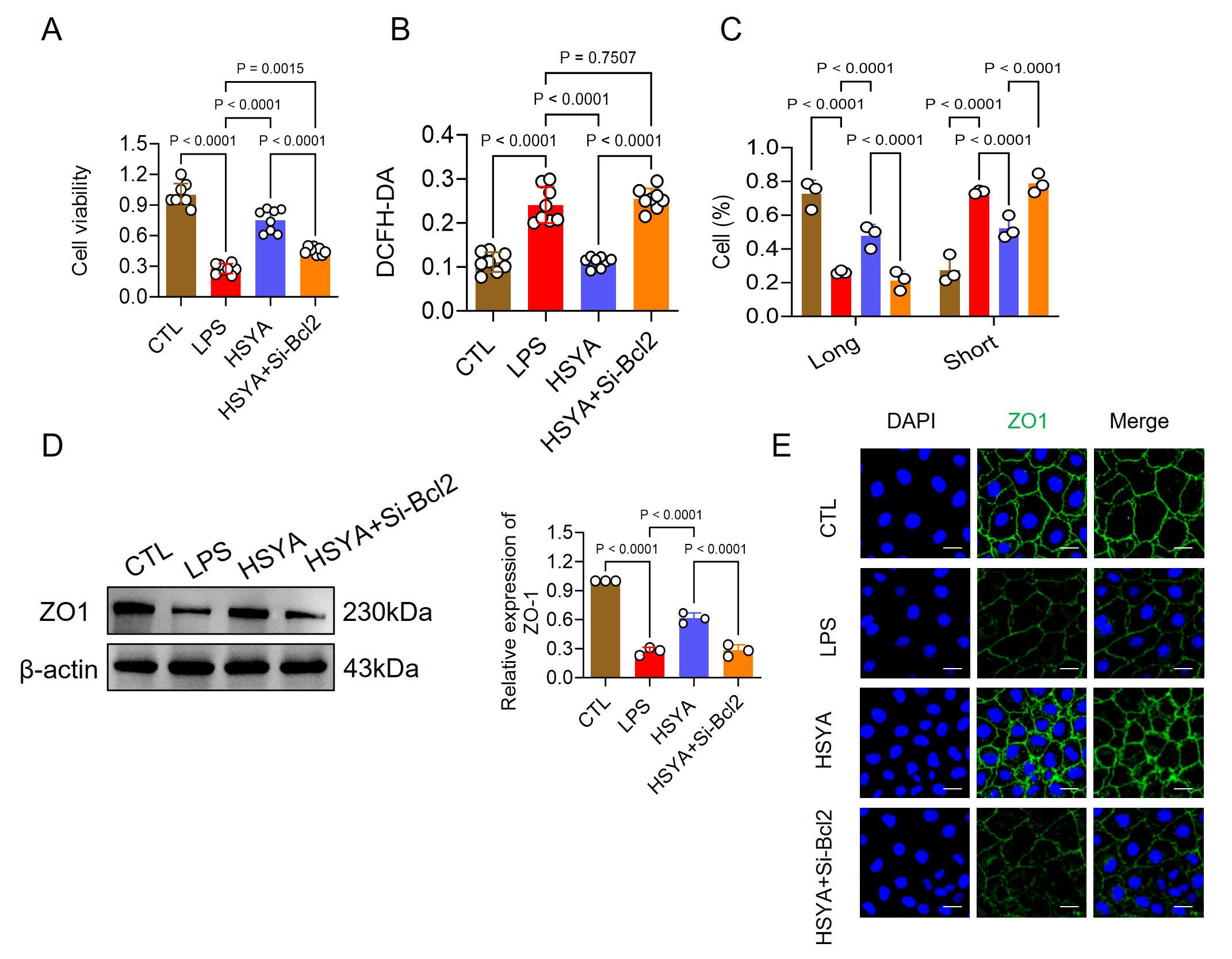

Supplement: Supplementary file 3 [file Image2.jpeg]
